# Association between depression and dysmenorrhea among adolescent girls: multiple mediating effects of binge eating and sleep quality

**DOI:** 10.1186/s12905-023-02283-6

**Published:** 2023-03-28

**Authors:** Yingzhen Li, Baixue Kang, Xueyan Zhao, Xuena Cui, Jie Chen, Lijie Wang

**Affiliations:** 1 Department of Maternal and Child Health, School of Public Health, Cheeloo College of Medicine, Shandong University, Jinan, 250012 Shandong China; 2 Department of Obstetrics and Gynecology, Qilu Hospital, Shandong University, Jinan, 250012 Shandong China

**Keywords:** Adolescence, Dysmenorrhea, Menstrual cramps, Binge eating, Sleep quality, Depression

## Abstract

**Background:**

Dysmenorrhea has a significant negative impact on teenagers’ quality of life, and its prevalence is increasing annually. Although studies have explored the factors affecting dysmenorrhea, it remains unclear how these factors interact with one another. This study aimed to explore the mediating role of binge eating and sleep quality between depression and dysmenorrhea.

**Methods:**

This cross-sectional study recruited adolescent girls from the Health Status Survey of adolescents in Jinan, Shandong Province, and used multistage stratified cluster random sampling. Data was collected using an electronic questionnaire between March 9, 2022, and June 20, 2022. The Numerical Rating Scale and Cox Menstrual Symptom Scale were used to assess dysmenorrhea and the Patient Health Questionnaire-9 to assess depression. The mediation model was tested by Mplus 8.0, and the mediating effect was analyzed using the Product of Coefficients approach and the Bootstrap method.

**Results:**

Among the total of 7818 adolescent girls included in this study, the prevalence of dysmenorrhea is 60.5%. A significant positive association was found between dysmenorrhea and depression. Binge eating and sleep quality seemingly mediate this association. The mediating effect of sleep quality (21.31%) was greater than that of binge eating (6.18%).

**Conclusions:**

The findings of this study point in the right direction for preventing and treating dysmenorrhea in adolescents. For adolescent dysmenorrhea, mental health should be considered and proactive steps taken for educating adolescents on healthy lifestyles to reduce negative consequences of dysmenorrhea. Longitudinal studies on the causal link and influence mechanisms between depression and dysmenorrhea should be conducted in the future.

**Supplementary Information:**

The online version contains supplementary material available at 10.1186/s12905-023-02283-6.

## Introduction

Dysmenorrhea is a type of pain that includes symptoms such as lower abdominal cramps and other discomfort before and/or during menstruation [1]. It can be divided into primary and secondary dysmenorrhea [2]. Primary dysmenorrhea is characterized by lower abdominal pain without organic lesions, whereas secondary dysmenorrhea is caused by organic lesions in the pelvis [3]. A series of physiological-psychological-nerve-endocrine changes occur in adolescence [4], and menstrual health specifically is affected by complex physiological and psychological changes [5]. According to a previous study, dysmenorrhea has the highest prevalence (89.7%) of menstrual disorders [6]. The prevalence of dysmenorrhea in adolescents varies greatly across the globe, with previous studies indicating a prevalence ranging from 20%-90% [7]. For example, the prevalence of dysmenorrhea in adolescents is 77.8% in Osogbo [8], 38.1% in Lebanon [9], and 89% in Sweden [10]. In recent years, 16%–93% of adolescent girls reported dysmenorrhea [11]. Dysmenorrhea has a direct adverse impact on the quality of life [11]; for instance, dysmenorrhea affects women’s social lives and students’ academic performance due to issues including absenteeism and inability to participate in physical activities [12].

Studies have shown that negative emotions may aggravate dysmenorrhea [13], but the mechanism is not completely clear. Depression is a common negative emotion. During the epidemic period of the coronavirus disease 2019 (COVID-19), the incidence of depression increased and was much higher in girls (14.65%) than in boys (9.04%) [14–16]. Furthermore, studies have shown that the frequency of depressive symptoms after SARS-CoV-2 infection ranges from 11 to 28% [17]. Therefore, it is necessary to further explore the possible mechanisms between depression and dysmenorrhea for more comprehensive dysmenorrhea management and treatment.

According to the psychodynamic hypothesis, behavior is primarily influenced by psychological forces, leading to the development of physical symptoms [18, 19]. Behavioral psychology theory points out that implicit psychology predominates over explicit actions because the main objective of psychology is to predict and control behavior [20]. Therefore, we hypothesized that depression may trigger dysmenorrhea through certain behavioral factors. Previous research has shown that eating behavior can mediate the association between depression and other diseases such as obesity and cardiovascular disease [21, 22]. Moreover, a school-based study in Australia found that 12.4% of adolescents reported binge eating at least once per week [23]. Another study in Bahrain showed that 21.2% of young people engaged in binge eating [24]. However, no study has examined whether dietary behaviors mediate the association between depression and dysmenorrhea. In addition, a study has shown that 52.7% of adolescents have poor sleep quality [25]. During adolescence, the prevalence of sleep disorders is higher in girls than in boys [26]. Sleep quality has a partially mediated effect on depression and pain [27]; however, it is unclear whether this relationship holds true for dysmenorrhea.

Individuals with binge eating behaviors reportedly have hypersensitivity to certain interoceptive signals [28]. Therefore, binge eating may aggravate the discomfort associated with dysmenorrhea. Depression is related to eating disorders [29], and even predicts eating disorders during adolescence [30]; therefore, we hypothesized that depression can indirectly affect dysmenorrhea through binge eating behavior. To date, studies have found no significant association between dysmenorrhea and factors including sleep work patterns and sleep time, but there is a significant association between dysmenorrhea and sleep quality [31]. Students with depression were 2.47 times more likely to develop sleep disorders than other students [32]. Therefore, we hypothesized that depression could indirectly affect dysmenorrhea by affecting sleep quality. Additionally, excessive carbohydrate intake is harmful to sleep quality [33], therefore, based on psychodynamic and behavioral psychology theories, we hypothesized that depression can indirectly affect sleep quality through binge eating behavior.

Depression, binge eating behavior, and poor sleep quality are all risk factors for dysmenorrhea; however, no study has identified the interactional mechanisms between these risk factors. In previous studies, the diagnostic criteria for dysmenorrhea were different [34], and there are few studies with multicenter, large samples reporting dysmenorrhea in adolescents since the COVID-19 outbreak. Therefore, our research is devoted to addressing the following problems:

1. The current prevalence of dysmenorrhea among adolescent girls;
2. The correlation between depression and dysmenorrhea; and
3. The mediating effects of binge eating and sleep quality on depression and dysmenorrhea.

## Materials and methods

### Participants

Data were collected from a cross-sectional study conducted in Jinan, Shandong Province, from March 9, 2022, to June 20, 2022, that aimed to explore the health status of adolescents. The respondents were junior and senior high school students. Information on sociodemographic characteristics, mental health, lifestyle, and menstruation-related variables were included. The menstruation-related questions were only asked for girls to answer. We use the stratified multistage random sampling to select participants from all districts and counties in Jinan (10 districts and 2 counties). In the first stage, according to the probability proportionate-to-size sampling method (PPS) [35], 1 junior high school and 1 senior high school were randomly selected from 10 districts, 3 junior high schools and 1 senior high school from 2 counties, and a total of 6 vocational high schools from all districts and counties of Jinan. A total of 16 junior, 12 senior, and 6 vocational high schools were selected. In the second stage, based on the PPS, 3–5 classes were selected separately from each grade of each school, and a total of 443 classes were selected. Lastly, all students in the selected class completed an electronic questionnaire. In the Health Status Survey of Adolescents in Jinan, Shandong Province, a total of 17,703 questionnaires were collected, of which 8,685 were girls and 9018 were boys, for a response rate of 86.7%. In this study, the 8685 girls from the Health Status Survey of Adolescents were included. Information about the Cox Menstrual Symptom Scale (CMSS), the Patient Health Questionnaire-9 (PHQ-9), binge eating, sleep quality, and sociodemographic characteristics from the questionnaire were all incorporated into this study. The inclusion criterion for this study was middle and high school girls aged 10 to 20 years; the exclusion criterion was students who had not yet reached menarche. In addition, questionnaires with incomplete answers were discarded during the data analysis process. In total, 7818 eligible female respondents were included in this study. The effectiveness rate of the questionnaire was 90.02%.

The Public Health Ethics Committee of Shandong University reviewed and approved the study protocol (approval number: LL20211116). All participants provided informed consent.

### Measures

#### Dysmenorrhea

The severity and duration of dysmenorrhea symptoms were evaluated using the CMSS (see Supplementary Table 1). The scale was developed by Professor Daniel J. Cox in 1978 to comprehensively evaluate the severity and duration of dysmenorrhea symptoms [36]. The Cronbach's alpha coefficient of the Cox Menstrual Symptom Scale of the Chinese version was 0.833 and the KMO was 0.811 [37]. This scale contains 18 items, each with two assessment dimensions: severity and duration of dysmenorrhea symptoms. The 18 items include: “general aching, headaches, stomachache, backaches, cramps, leg aches, dizziness, facial blemishes, flushing, nausea, vomiting, loss of appetite, diarrhea, weakness, insomnia, gloomy, irritability, and nervousness.” In the severity level assessment, each symptom was scored on a 5-point scale: 0 = no discomfort, 1 = mild discomfort, 2 = moderate discomfort, 3 = severe discomfort, and 4 = very severe discomfort. In the duration level assessment, each symptom was also scored on a 5-point scale: 0 = none, 1 = lasting 0–3 h, 2 = lasting 3–7 h, 3 = lasting 7–24 h, and 4 = lasting > 24 h. The total score of the two dimensions can be calculated to comprehensively evaluate dysmenorrhea symptoms. The total score ranges from 0 to 144. The higher the score, the more severe the dysmenorrhea. In this study, the Cronbach's alpha coefficient of the Cox Menstrual Symptom Scale was 0.963 and the KMO was 0.897.

Using the Numerical Rating Scale (NRS) to classify the severity of dysmenorrhea. According to previous research, the NRS has been widely used in various studies to assess the degree of pain, and it has high reliability and validity [38]. Dysmenorrhea was categorized into 11 levels: 0 = no dysmenorrhea, 1–3 = mild pain, 4–6 = moderate pain, 7–9 = severe pain, and 10 = very severe pain.

#### Depression

The depression symptoms in the past 2 weeks were assessed using the PHQ-9 (see Supplementary Table 2), a study has confirmed that the PHQ-9 is a reliable and valid measure of depression [39]. The questionnaire consisted of nine items: “1. Little interest or pleasure in doing things.” “2. Feeling down, depressed, or hopeless.” “3. Trouble falling or staying asleep, or sleeping too much.” “4. Feeling tired or having little energy.” “5. Poor appetite or overeating.” “6. Feeling bad about yourself—or that you are a failure or have let yourself or your family down.” “7. Trouble concentrating on things, such as reading the newspaper or watching television.” “8. Moving or speaking so slowly that other people could have noticed, or the opposite—being so fidgety or restless that you have been moving around a lot more than usual.” “9. Thoughts that you would be better off dead or of hurting yourself in some way.” Each item rated on a 4-point scale (0 = never, 1 = for a few days, 2 = just over half of the days, and 3 = almost daily). The total score ranged from 0 to 27, with a total score of 0–4 indicating no depression, 5–9 indicating mild depression possibly, 10–14 indicating moderate depression possibly, 15–19 indicating moderate-to-severe depression possibly; and 20–27 indicating severe depression possibly. In this study, Cronbach’s alpha coefficient of the PHQ-9 was 0.925 and the KMO was 0.945.

#### Mediating variables

Mediating variables included binge eating and sleep quality. Binge eating is when you eat more food than the majority of people do in comparable circumstances and you are unable to stop, regulate what you eat, or regulate how much you eat [40]. Sleep quality refers to how satisfied you are with your sleeping experience. Good sleep quality means that you feel rested and refreshed. You enjoy your sleep and have fulfilling familial and social interactions [41]. Binge eating was classified as frequent, occasional, or never, and sleep quality was classified as good, general, or poor.

#### Covariates

We controlled for age (10–13 years, 14–17 years, 18–20 years), school type (junior high school, senior high school, vocational high school), parental education level (junior high school and below, senior high school/technical secondary school, bachelor’s/college degree and above), boarding at school (yes = 1, no = 0), maternal history of dysmenorrhea (unclear, yes, no), and average sleep duration per night (excessive, normal, inadequate).

### Statistical analysis

Statistical analysis was performed using SPSS version 26.0 and Mplus version 8.0. We tested whether the data followed a normal distribution before analysis. We present sample characteristics as frequency (percentage). Nonparametric tests (Kruskal–Wallis test and Mann–Whitney U test) were performed to compare the total CMSS scale scores in subgroups of different categorical variables. In addition, the main study variables (depression level, binge eating, sleep quality, and dysmenorrhea symptoms) were tested using Spearman’s rank correlation.

Finally, Mplus version 8.0 was used to test the mediating effect of binge eating and sleep quality on depression and dysmenorrhea. A significant mediation effect was demonstrated if the 95% confidence intervals (CIs) of the interaction did not contain 0 (effect test *p* < 0.05), and 95% bias-corrected CIs was yielded by 5,000 bootstrap estimates. Statistical significance was defined as a two-tailed *p*-value of < 0.05.

## Results

### Sample characteristics

The mean age of the 7,818 adolescent girls included in this study was 15.71 ± 1.617 years. The prevalence of dysmenorrhea is 60.5% (*n* = 4732), and that of mild, moderate, severe, and very severe dysmenorrhea was 12.8%, 33.7%, 12.4%, and 1.6%, respectively. In addition, information about the frequency of CMSS and PHQ-9 are shown in Supplementary Table 3 and  4.

Table 1 summarizes the sociodemographic information of the 4,732 girls with dysmenorrhea and the univariate analysis of the total CMSS scale score in the subgroups of different category variables. Univariate analysis showed that age, type of school, boarding at school, maternal history of dysmenorrhea, sleep duration, sleep quality, and binge eating were significantly associated with dysmenorrhea.

**Table 1:** Characteristics of the sample (*N* = 4732)

**Variables**	**N(%)**	**CMSS Score**	**H/Z**	**P**
		**M(P25-P75)**		
Observation variable	4732(100.0)	27.0(14.00–45.00)		
Age			59.259^a^	0.000
10–13	394(8.3)	19.0(8.75–34.00)		
14–17	3611(76.3)	27.0(14.00–46.00)		
18–20	727(15.4)	30.0(15.00–48.00)		
Type of school			173.956^a^	0.000
Junior high school	1776(37.5)	21.0(10.00–37.00)		
Vocational high school	844(17.8)	33.0(18.00–56.00)		
Senior high school	2112(44.6)	30.0(15.25–48.00)		
Father's education			0.586^a^	0.746
Junior high school and below	2371(50.1)	27.0(14.00–45.00)		
Senior high school	1242(26.2)	26.0(13.00–45.00)		
Bachelor’s/college degree and above	1119(23.6)	27.0(14.00–45.00)		
Mother’s education			3.582^a^	0.167
Junior high school and below	2677(56.6)	26.0(13.00–45.00)		
Senior high school	1101(23.2)	26.0(13.00–45.00)		
Bachelor’s/college degree and above	954(20.2)	29.0(14.00–46.00)		
Boarding at school			2.198^b^	0.028
Yes	2130(45.0)	28.0(14.00–47.00)		
No	2602(55.0)	26.0(14.00–44.00)		
Mother's history of dysmenorrhea			49.071^a^	0.000
Yes	2305(48.7)	29.0(16.00–48.00)		
No	1050(22.2)	23.0(10.00–41.00)		
No clear	1377(29.1)	27.0(14.00–45.00)		
Sleep duration			61.692^a^	0.000
Inadequate	3348(70.8)	29.0(15.00–47.00)		
Normal	1351(28.6)	23.0(10.00–40.00)		
Excessive	33(0.7)	30.0(21.00–59.50)		
Sleep quality			906.332^a^	0.000
Good	1953(41.3)	17.0(8.00–32.00)		
General	2195(46.4)	31.0(18.00–46.00)		
Poor	584(12.3)	55.0(36.00–76.00)		
Binge eating			265.551^a^	0.000
Often	126(2.7)	43.0(19.75–69.00)		
Sometimes	1223(25.8)	36.0(21.00–56.00)		
Never	3383(71.5)	23.0(11.00–40.00)		

*CMSS* the Cox Menstrual Symptom Scale

^a^Kruskal-wallis test

^b^Mann-Whitney U test

### Preliminary analyses

Table 2 presents the Spearman’s rank correlation coefficients for the main study variables. Depression was positively associated with dysmenorrhea (*r* = 0.543, *p* < 0.01), binge eating behavior (*r* = 0.262, *p* < 0.01), and poor sleep quality (*r* = 0.334, *p* < 0.01). Binge eating behavior was positively associated with dysmenorrhea (*r* = 0.237, *p* < 0.01) and poor sleep quality (*r* = 0.112, *p* < 0.01). Poor sleep quality was positively associated with dysmenorrhea (*r* = 0.428, *p* < 0.01).

**Table 2:** The correlations among the main study variables

**Variables**	**1**	**2**	**3**	**4**
Depression score	1			
CMSS score	0.543^**^	1		
Binge eating	0.262^**^	0.237^**^	1	
Sleep quality	0.334^**^	0.428^**^	0.112^**^	1

*CMSS* the Cox Menstrual Symptom Scale

^**^ *p* < 0.01

### Testing for the mediating effect

The mediator model showed an acceptable fit: χ^2^/df = 1.705, CFI = 0.994, TLI = 0.990, RMSEA = 0.012, and SRMR = 0.059.

**Fig. 1:**
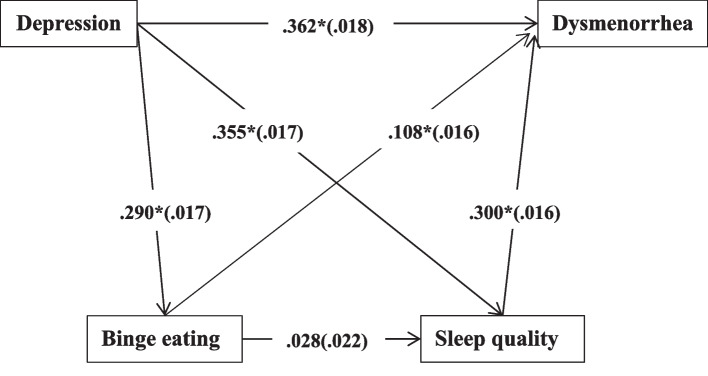
Mediating effects of binge eating and sleep quality on depression and dysmenorrhea. Note: adjusted for age, school type, educational level of parents, boarding at school, mother's history of dysmenorrhea, and sleep duration. Standardized pathway coefficients are shown outside in parentheses, and standard errors are shown in parentheses, * *p* < 0.01

As shown in Table 3 and Fig. 1, binge eating and sleep quality had partial mediating effects on depression and dysmenorrhea, respectively. The difference in the mediating effects between binge eating and sleep quality was statistically significant (Table 4), and the mediating effect of sleep quality (21.31%) was greater than that of binge eating (6.18%); however, the binge eating to sleep quality pathway was not statistically significant (*P* = 0.213).

**Table 3:** The chained mediation model and 95% CIs

**Model pathways**	**Point** **Estimate**	**Product of Coefficients**			**Bootstrap 5000 Times**	
					**95%CI**	
		**S.E**	**Est./S.E**	***P* Value**	**Lower**	**Upper**
Path A	0.107	0.008	13.644	0.000	0.092	0.122
Path B	0.031	0.005	6.217	0.000	0.022	0.042
Path C	0.002	0.002	1.245	0.213	-0.002	0.006
Path D	0.362	0.018	20.475	0.000	0.323	0.391
Total indirect	0.140	0.009	15.518	0.000	0.123	0.158
Total	0.502	0.015	34.542	0.000	0.467	0.565

Adjusted for age, school type, educational level of parents, boarding at school, mother's history of dysmenorrhea, and sleep duration. The values listed in the table are all standardized values. *S.E* Standard error

Path A: Depression- > Sleep Quality- > Dysmenorrhea

Path B: Depression- > Binge Eating- > Dysmenorrhea

Path C: Depression- > Binge Eating- > Sleep quality- > Dysmenorrhea

Path D: Depression- > Dysmenorrhea

**Table 4:** The parallel mediation, 95% CIs and comparison between the mediation effects

**Model pathways**	**Point** **Estimate**	**Product of Coefficients**			**Bootstrap 5000 Times**	
					**95%CI**	
		**S.E**	**Est./S.E**	***P* Value**	**Lower**	**Upper**
**Indirect Effects**						
Path AB1	0.153	0.023	6.633	0.000	0.112	0.205
Path AB2	0.500	0.035	14.205	0.000	0.436	0.575
Total indirect	0.653	0.043	15.063	0.000	0.578	0.747
Direct	1.626	0.080	20.225	0.000	1.464	1.775
Total	2.279	0.047	32.135	0.000	2.137	2.418
**Contrast**						
Path AB1 vs. Path AB2	-0.347	0.041	-8.491	0.000	-0.428	-0.270

Note: adjusted for age, school type, educational level of parents, boarding at school, mother's history of dysmenorrhea, and sleep duration. The values listed in the table are all non-standardized values. *S.E* Standard error

Path AB1: Depression- > Binge eating- > Dysmenorrhea

Path AB2: Depression- > Sleep Quality- > Dysmenorrhea

## Discussion

More than half of the adolescent girls in this study experienced dysmenorrhea, with more than one-third of those experiencing moderate dysmenorrhea. This is inconsistent with the results of the prior study, which found that the prevalence of mild dysmenorrhea had the highest proportion [31]. Although the overall prevalence in our study is high, it is lower than the results of said previous study [31]. A possible reason for this is that there are no uniform diagnostic criteria for dysmenorrhea. Additionally, it might be because the COVID-19 pandemic has affected the prevalence of dysmenorrhea in teenagers in recent years.

The current study focused on the relationship between depression and dysmenorrhea in adolescent girls and revealed the possible mechanisms underlying the relationship between depression and dysmenorrhea through a mediation model. We found a positive association between depression and dysmenorrhea. Binge eating and sleep quality had a partial mediating effect on depression and dysmenorrhea, respectively. The mediating effect of sleep quality (21.31%) was greater than that of binge eating (6.18%).

Three possible pathways may explain the association between depression and dysmenorrhea. In the physiological pathway, we hypothesize that because depression and other bad emotions can result in psychological imbalance and neuroendocrine abnormalities, which in turn stimulate the uterus, increased uterine isthmus tension can either cause or worsen dysmenorrhea. Studies have shown that the monoamine hypothesis of depression states that the neurotransmitter-serotonin release function is weakened in the brains of patients with depression [42], and serotonin release can participate in the inhibition of some types of pain [43]. Therefore, patients with depression may be more sensitive to dysmenorrhea. In the behavioral pathway, we mainly considered dietary behavior, which was represented by binge eating. In the sleep pathway, we primarily considered sleep quality.

Our results show that the association between depression and dysmenorrhea is partially mediated by binge eating, probably revealing one of the dietary behavioral mechanisms regarding how depressive symptoms indirectly affect dysmenorrhea symptoms. We think that one of the ways that depression indirectly influences dysmenorrhea through binge eating is that depressed people may prefer to eat to soothe an unpleasant mood, and that both binge eating and depression may lead to endocrine abnormalities that cause or worsen dysmenorrhea. A previous study has shown that the gray matter volume increases in the left anterior abdominal insula of people with binge eating behavior, causing hypersensitivity [44], which may aggravate the uncomfortable feeling of dysmenorrhea. Depressed people have elevated cortisol levels [45], elevated cortisol levels increase food intake [46], which may contribute to the significant positive relationship between depression and binge eating behavior.

Our results also showed that the association between depression and dysmenorrhea is partially mediated by sleep quality, probably revealing one of the sleep mechanisms regarding how depressive symptoms indirectly affect dysmenorrhea symptoms. We believe that sleep problems are almost always a common sign of depression and that poor sleep quality is likely to interfere with the production of prostaglandins, which can cause dysmenorrhea. Experiments have shown that prefrontal density declines in the brains of individuals with depression. In this region, genes that control circadian rhythms regulate sleep dysregulation [47]. The effect of sleep quality on dysmenorrhea may be due to overlapping central nervous system mechanisms [48]. In this study, the path coefficient for binge eating-sleep quality was not significant. However, previous research found a link between dietary habits and sleep problems [33]. It is possible that other mechanisms influence the relationship between dietary behaviors and sleep disorders, and further research is required to clarify this relationship.

Our study has several public health implications. Government can implement measures to prevent and relieve dysmenorrhea in adolescents by focusing on the prevention and treatment of depression and educating teenagers to develop good living habits. Firstly, relevant personnel should pay attention to the psychological problems of teenagers, especially in the context of the normalization of the COVID-19 epidemic because they frequently arise in this context [49]. Teenagers are an especially vulnerable group, relevant departments should pay more attention to their mental health and provide necessary psychological support services. Secondly, educators need to correct students' living habits, to maintain good eating behaviors, especially educate young people in developing good sleep habits.

To the best of our knowledge, few studies have the sample size included in this study. For the first time, our study examined the interaction mechanisms of psychological factors (depressive symptoms), dietary behavioral factors (binge eating behavior), and sleep problems (sleep quality) on dysmenorrhea in adolescents. Most previous studies of dysmenorrhea focused only on the unidirectional dimension of the severity of lower abdominal pain, whereas the CMSS covers a broader range of dysmenorrhea symptoms and dimensions.

The limitations of our study can provide directions for further research. Firstly, the data collection with a structured self-report questionnaire could not exclude the recall bias. Secondly, the cross-sectional study examined only the relationship between depression and dysmenorrhea, making it difficult to conclude causal inferences between depression and dysmenorrhea. Furthermore, using a self-report questionnaire rather than a clinical diagnosis, the judgment of dysmenorrhea may not be completely correct, and there is no way to distinguish between primary dysmenorrhea and secondary dysmenorrhea. In the future, a combination of clinical diagnostic techniques will be needed to distinguish between primary and secondary dysmenorrhea for further research. Finally, this study can only represent the situation of adolescent girls in Jinan City, Shandong Province; whether the conclusions can be generalized to other regions is unknown. Further longitudinal studies should be conducted on the causal relationship and mechanism of influence between depression and dysmenorrhea. Multicenter, large-sample research should be conducted in the future. Simultaneously, investigators should combine public health technology with clinical diagnostic techniques.

## Conclusion

Sleep quality and binge eating partially mediate the positive association between depression and dysmenorrhea. The mediating effect of sleep quality (21.31%) was greater than that of binge eating (6.18%). Our results encourage educators to concentrate on the mental health problems of adolescents while educating them to develop good life habits to prevent and relieve dysmenorrhea symptoms, further improving the quality of life of adolescents. In the future, longitudinal studies and multicenter, large-sample research should be conducted. Simultaneously, we should combine public health technology with clinical diagnostic techniques.

## Supplementary Information

**Additional file 1: Supplementary Table 1.** The Cox Menstrual Symptom Scale. **Supplementary Table 2.** The Patient Health Questionnaire-9. **Supplementary Table 3.** Distribution of frequency of the CMSS. **Supplementary Table 4.** Distribution of frequency of the Questions in PHQ-9.

## Data Availability

The data are available on request from the corresponding author in this study.

## References

[1] Kabukçu C, Kabukçu Başay B, Başay Ö (2021). Primary dysmenorrhea in adolescents: Association with attention deficit hyperactivity disorder and psychological symptoms. Taiwan J Obstet Gynecol.

[2] Nagy H, Khan MAB (2022). Dysmenorrhea. 2022 Jul 18. StatPearls.

[3] Gutman G, Nunez AT, Fisher M (2022). Dysmenorrhea in adolescents. Curr Probl Pediatr Adolesc Health Care.

[4] Marshall WA, Tanner JM (1969). Variations in pattern of pubertal changes in girls. Arch Dis Child.

[5] Rigon F, De Sanctis V, Bernasconi S, Bianchin L, Bona G, Bozzola M, Buzi F, Radetti G, Tatò L, Tonini G, De Sanctis C, Perissinotto E (2012). Menstrual pattern and menstrual disorders among adolescents: an update of the Italian data. Ital J Pediatr.

[6] Rafique N, Al-Sheikh MH (2018). Prevalence of menstrual problems and their association with psychological stress in young female students studying health sciences. Saudi Med J.

[7] Davis AR, Westhoff CL (2001). Primary dysmenorrhea in adolescent girls and treatment with oral contraceptives. J Pediatr Adolesc Gynecol.

[8] Amu EO, Bamidele JO (2014). Prevalence of menstrual disorders among adolescent girls in Osogbo, South Western Nigeria. Int J Adolesc Med Health.

[9] Karout N, Hawai SM, Altuwaijri S (2012). Prevalence and pattern of menstrual disorders among Lebanese nursing students. East Mediterr Health J.

[10] Söderman L, Edlund M, Marions L (2019). Prevalence and impact of dysmenorrhea in Swedish adolescents. Acta Obstet Gynecol Scand.

[11] De Sanctis V, Soliman A, Bernasconi S, Bianchin L, Bona G, Bozzola M, Buzi F, De Sanctis C, Tonini G, Rigon F, Perissinotto E (2015). Primary Dysmenorrhea in Adolescents: Prevalence, Impact and Recent Knowledge. Pediatr Endocrinol Rev.

[12] Iacovides S, Avidon I, Baker FC (2015). What we know about primary dysmenorrhea today: a critical review. Hum Reprod Update..

[13] Mou L, Lei W, Chen J, Zhang R, Liu K, Liang X (2019). Mediating effect of interpersonal relations on negative emotions and dysmenorrhea in female adolescents. Gen Psychiatr.

[14] Safi-Keykaleh M, Aliakbari F, Safarpour H, Safari M, Tahernejad A, Sheikhbardsiri H, Sahebi A (2022). Prevalence of postpartum depression in women amid the COVID-19 pandemic: A systematic review and meta-analysis. Int J Gynaecol Obstet.

[15] Heidarijamebozorgi M, Jafari H, Sadeghi R, Sheikhbardsiri H, Kargar M, Gharaghani MA (2021). The prevalence of depression, anxiety, and stress among nurses during the coronavirus disease 2019: a comparison between nurses in the frontline and the second line of care delivery. Nurs Midwifery Stud..

[16] Chen F, Zheng D, Liu J, Gong Y, Guan Z, Lou D (2020). Depression and anxiety among adolescents during COVID-19: A cross-sectional study. Brain Behav Immun.

[17] Renaud-Charest O, Lui LMW, Eskander S, Ceban F, Ho R, Di Vincenzo JD, Rosenblat JD, Lee Y, Subramaniapillai M, McIntyre RS (2021). Onset and frequency of depression in post-COVID-19 syndrome: A systematic review. J Psychiatr Res.

[18] Gallop R, O'Brien L (2003). Re-establishing psychodynamic theory as foundational knowledge for psychiatric/mental health nursing. Issues Ment Health Nurs.

[19] Yoshihara K, Kubo C (2009). Psychosomatic disorder and functional somatic syndrome. Nihon Rinsho..

[20] de Freitas AS, Saraiva FT, de Carvalho Neto MB (2019). Reevaluating the initial impact of John Broadus Watson on American psychology: The necessity of comparative parameters. J Hist Behav Sci.

[21] Kang J, Moser DK, Biddle MJ, Lennie TA, Smyth SS, Vsevolozhskaya OA (2020). Inflammatory properties of diet mediate the effect of depressive symptoms on Framingham risk score in men and women: Results from the National Health and Nutrition Examination Survey (2007–2014). Nutr Res.

[22] Darbandi M, Najafi F, Pasdar Y, Rezaeian S (2020). Structural equation model analysis for the evaluation of factors associated with overweight and obesity in menopausal women in RaNCD cohort study. Menopause.

[23] Bentley C, Gratwick-Sarll K, Harrison C, Mond J (2015). Sex differences in psychosocial impairment associated with eating disorder features in adolescents: A school-based study. Int J Eat Disord.

[24] Abdulla ZARA, Almahmood HO, Alghasra RR, Alherz ZAS, Alsharifa HAG, Qamber SJ, Alomar NA, Almajed FE, Almahroos TR, Alnajjas ZA, Alsayyad AS (2023). Prevalence and associated factors of binge eating disorder among Bahraini youth and young adults: a cross-sectional study in a self-selected convenience sample. J Eat Disord.

[25] Lemma S, Patel SV, Tarekegn YA, Tadesse MG, Berhane Y, Gelaye B, Williams MA (2012). The Epidemiology of Sleep Quality, Sleep Patterns, Consumption of Caffeinated Beverages, and Khat Use among Ethiopian College Students. Sleep Disord.

[26] Wang Z, Dang J, Zhang X, Moore JB, Li R (2021). Assessing the relationship between weight stigma, stress, depression, and sleep in Chinese adolescents. Qual Life Res.

[27] Nephew BC, Incollingo Rodriguez AC, Melican V, Polcari JJ, Nippert KE, Rashkovskii M, Linnell LB, Hu R, Ruiz C, King JA, Gardiner P (2022). Depression Predicts Chronic Pain Interference in Racially Diverse Income-Disadvantaged Patients. Pain Med.

[28] Khalsa SS, Lapidus RC (2016). Can Interoception Improve the Pragmatic Search for Biomarkers in Psychiatry?. Front Psychiatry.

[29] Lopez-Aguilar I, Ibarra-Reynoso LDR, Malacara JM (2018). Association of Nesfatin-1, Acylated Ghrelin and Cortisol with Scores of Compulsion, Food Addiction, and Binge Eating in Adults with Normal Weight and with Obesity. Ann Nutr Metab.

[30] Ferreiro F, Seoane G, Senra C (2012). Gender-related risk and protective factors for depressive symptoms and disordered eating in adolescence: a 4-year longitudinal study. J Youth Adolesc.

[31] Liu X, Chen H, Liu ZZ, Fan F, Jia CX. Early Menarche and Menstrual Problems Are Associated with Sleep Disturbance in a Large Sample of Chinese Adolescent Girls. Sleep. 2017;40(9):1–11. 10.1093/sleep/zsx107 28645184

[32] Guo L, Deng J, He Y, Deng X, Huang J, Huang G, Gao X, Lu C (2014). Prevalence and correlates of sleep disturbance and depressive symptoms among Chinese adolescents: a cross-sectional survey study. BMJ Open.

[33] Afaghi A, O'Connor H, Chow CM (2007). High-glycemic-index carbohydrate meals shorten sleep onset. Am J Clin Nutr.

[34] Dmitrović R (2000). Transvaginal color Doppler study of uterine blood flow in primary dysmenorrhea. Acta Obstet Gynecol Scand.

[35] Qin W, Xu L, Wu S, Shao H (2021). Income, Relative Deprivation and the Self-Rated Health of Older People in Urban and Rural China. Front Public Health.

[36] Cox DJ, Meyer RG (1978). Behavioral treatment parameters with primary dysmenorrhea. J Behav Med.

[37] Ma YX, Ma HY, Chen SZ, Gao SZ (2015). Reliability and validity of Chinese version of Cox dysmenorrhea symptom scale. J Shandong Univ Tradit Chin Med.

[38] de Arruda GT, Driusso P, Rodrigues JC, de Godoy AG, Avila MA (2022). Numerical rating scale for dysmenorrhea-related pain: a clinimetric study. Gynecol Endocrinol.

[39] Kroenke K, Spitzer RL, Williams JB (2001). The PHQ-9: validity of a brief depression severity measure. J Gen Intern Med.

[40] Waller D (2001). Binge eating. BMJ.

[41] Nelson KL, Davis JE, Corbett CF (2022). Sleep quality: An evolutionary concept analysis. Nurs Forum.

[42] Hirschfeld RM (2000). History and evolution of the monoamine hypothesis of depression. J Clin Psychiatry.

[43] Paredes S, Cantillo S, Candido KD, Knezevic NN (2019). An Association of Serotonin with Pain Disorders and Its Modulation by Estrogens. Int J Mol Sci.

[44] Murray SB, Duval CJ, Balkchyan AA, Cabeen RP, Nagata JM, Toga AW, Siegel SJ, Jann K (2022). Regional gray matter abnormalities in pre-adolescent binge eating disorder: A voxel-based morphometry study. Psychiatry Res.

[45] Vrshek-Schallhorn S, Doane LD, Mineka S, Zinbarg RE, Craske MG, Adam EK (2013). The cortisol awakening response predicts major depression: predictive stability over a 4-year follow-up and effect of depression history. Psychol Med.

[46] Rosenberg N, Bloch M, Ben Avi I, Rouach V, Schreiber S, Stern N, Greenman Y (2013). Cortisol response and desire to binge following psychological stress: comparison between obese subjects with and without binge eating disorder. Psychiatry Res.

[47] Gabbott PL, Rolls ET (2013). Increased neuronal firing in resting and sleep in areas of the macaque medial prefrontal cortex. Eur J Neurosci.

[48] Finan PH, Smith MT (2013). The comorbidity of insomnia, chronic pain, and depression: dopamine as a putative mechanism. Sleep Med Rev.

[49] Xie Y, Xu E, Al-Aly Z (2022). Risks of mental health outcomes in people with covid-19: cohort study. BMJ.

