# Supplementary material for: Association between depression and dysmenorrhea among adolescent girls: multiple mediating effects of binge eating and sleep quality
Source: BMC Womens Health. 2023 Mar 28;23:140. doi: 10.1186/s12905-023-02283-6 (PMC10043526; doi:10.1186/s12905-023-02283-6)
Supplement: Supplementary file 1 — Additional file 1: Supplementary Table 1. The Cox Menstrual Symptom Scale. Supplementary Table 2. The Patient Health Questionnaire-9. Supplementary Table 3. Distribution of frequency of the CMSS. Supplementary Table 4. Distribution of frequency of the Questions in PHQ-9. [file 12905_2023_2283_MOESM1_ESM.docx]

**Supplementary Table 1** The Cox Menstrual Symptom Scale.

| Items | Severity of dysmenorrhea symptoms | | | | |  | Duration of dysmenorrhea symptoms | | | | |
| --- | --- | --- | --- | --- | --- | --- | --- | --- | --- | --- | --- |
|  | None | Mild | Moderate | Severe | Very severe |  | None | 0–3 hours | 3–7 hours | 7–24 hours | >24 hours |
| 1. General aching | 0 | 1 | 2 | 3 | 4 |  | 0 | 1 | 2 | 3 | 4 |
| 2. Headaches | 0 | 1 | 2 | 3 | 4 |  | 0 | 1 | 2 | 3 | 4 |
| 3. Stomachache | 0 | 1 | 2 | 3 | 4 |  | 0 | 1 | 2 | 3 | 4 |
| 4. Backaches | 0 | 1 | 2 | 3 | 4 |  | 0 | 1 | 2 | 3 | 4 |
| 5. Cramps | 0 | 1 | 2 | 3 | 4 |  | 0 | 1 | 2 | 3 | 4 |
| 6. Leg aches | 0 | 1 | 2 | 3 | 4 |  | 0 | 1 | 2 | 3 | 4 |
| 7. Dizziness | 0 | 1 | 2 | 3 | 4 |  | 0 | 1 | 2 | 3 | 4 |
| 8. Facial blemishes | 0 | 1 | 2 | 3 | 4 |  | 0 | 1 | 2 | 3 | 4 |
| 9. Flushing | 0 | 1 | 2 | 3 | 4 |  | 0 | 1 | 2 | 3 | 4 |
| 10. Nausea | 0 | 1 | 2 | 3 | 4 |  | 0 | 1 | 2 | 3 | 4 |
| 11. Vomiting | 0 | 1 | 2 | 3 | 4 |  | 0 | 1 | 2 | 3 | 4 |
| 12. Loss of appetite | 0 | 1 | 2 | 3 | 4 |  | 0 | 1 | 2 | 3 | 4 |
| 13. Diarrhea | 0 | 1 | 2 | 3 | 4 |  | 0 | 1 | 2 | 3 | 4 |
| 14. Weakness | 0 | 1 | 2 | 3 | 4 |  | 0 | 1 | 2 | 3 | 4 |
| 15. Insomnia | 0 | 1 | 2 | 3 | 4 |  | 0 | 1 | 2 | 3 | 4 |
| 16. Gloomy | 0 | 1 | 2 | 3 | 4 |  | 0 | 1 | 2 | 3 | 4 |
| 17. Irritability | 0 | 1 | 2 | 3 | 4 |  | 0 | 1 | 2 | 3 | 4 |
| 18. Nervousness | 0 | 1 | 2 | 3 | 4 |  | 0 | 1 | 2 | 3 | 4 |

**Supplementary Table 2** The Patient Health Questionnaire-9.

| Items | Not at all | Several days | More than half the days | Nearly every day |
| --- | --- | --- | --- | --- |
| 1. Little interest or pleasure in doing things | 0 | 1 | 2 | 3 |
| 2. Feeling down, depressed, or hopeless | 0 | 1 | 2 | 3 |
| 3. Trouble falling or staying asleep, or sleeping too much | 0 | 1 | 2 | 3 |
| 4. Feeling tired or having little energy | 0 | 1 | 2 | 3 |
| 5. Poor appetite or overeating | 0 | 1 | 2 | 3 |
| 6. Feeling bad about yourself—or that you are a failure or have let yourself or your family down | 0 | 1 | 2 | 3 |
| 7. Trouble concentrating on things, such as reading the newspaper or watching television | 0 | 1 | 2 | 3 |
| 8. Moving or speaking so slowly that other people could have noticed? Or the opposite—being so fidgety or restless that you have been moving around a lot more than usual | 0 | 1 | 2 | 3 |
| 9. Thoughts that you would be better off dead or of hurting yourself in some way | 0 | 1 | 2 | 3 |

**Supplementary Table 3** Distribution of frequency of the CMSS.

|  |  | Frequency (percentage) | | | | | | | | | |
| --- | --- | --- | --- | --- | --- | --- | --- | --- | --- | --- | --- |
|  | Severity of dysmenorrhea symptoms | | | | |  | Duration of dysmenorrhea symptoms | | | | |
|  | None | Mild | Moderate | Severe | Very severe |  | None | 0–3 hours | 3–7 hours | 7–24 hours | >24 hours |
| 1. General aching | 4398 (56.3) | 1669 (21.3) | 1241 (15.9) | 393 (5.0) | 117 (1.5) |  | 4411 (56.4) | 1748 (22.4) | 1016 (13.0) | 422 (5.4) | 221 (2.8) |
| 1. Headaches | 6115 (78.2) | 1073 (13.7) | 438 (5.6) | 147 (1.9) | 45 (0.6) |  | 6210 (79.4) | 1041 (13.3) | 378 (4.8) | 124 (1.6) | 65 (0.8) |
| 1. Stomachache | 5702 (72.9) | 1044 (13.4) | 702 (9.0) | 247 (3.2) | 123 (1.6) |  | 5804 (74.2) | 1181 (15.1) | 524 (6.7) | 192 (2.5) | 117 (1.5) |
| 1. Backaches | 4203 (53.8) | 1445 (18.5) | 1174 (15.0) | 647 (8.3) | 349 (4.5) |  | 4310 (55.1) | 1507 (19.3) | 945 (12.1) | 561 (7.2) | 495 (6.3) |
| 1. Cramps | 3357 (42.9) | 1523 (19.5) | 1512 (19.3) | 918 (11.7) | 508 (6.5) |  | 3481 (44.5) | 1844 (23.6) | 1218 (15.6) | 719 (9.2) | 556 (7.1) |
| 1. Leg aches | 5854 (74.9) | 1074 (13.7) | 580 (7.4) | 211 (2.7) | 99 (1.3) |  | 5967 (76.3) | 1064 (13.6) | 479 (6.1) | 180 (2.3) | 128 (1.6) |
| 1. Dizziness | 6083 (77.8) | 1008 (12.9) | 476 (6.1) | 182 (2.3) | 69 (0.9) |  | 6221 (79.6) | 1021 (13.1) | 382 (4.9) | 124 (1.6) | 70 (0.9) |
| 1. Facial blemishes | 5283 (67.6) | 1422 (18.2) | 744 (9.5) | 248 (3.2) | 121 (1.5) |  | 5476 (70.0) | 1364 (17.4) | 603 (7.7) | 225 (2.9) | 150 (1.9) |
| 1. Flushing | 6785 (86.8) | 668 (8.5） | 271 (3.5) | 66 (0.8) | 28 (0.4) |  | 6872 (87.9) | 620 (7.9) | 231 (3.0) | 64 (0.8) | 31 (0.4) |
| 1. Nausea | 6392 (81.8) | 827 (10.6) | 395 (5.1) | 118 (1.5) | 86 (1.1) |  | 6512 (83.3) | 826 (10.6) | 312 (4.0) | 97 (1.2) | 71 (0.9) |
| 1. Vomiting | 6987 (89.4) | 486 (6.2) | 226 (2.9) | 75 (1.0) | 44 (0.6) |  | 7060 (90.3) | 497 (6.1) | 192 (2.5) | 52 (0.7) | 35 (0.4) |
| 1. Loss of appetite | 5023 (64.2) | 1535 (19.6) | 825 (10.6) | 273 (3.5) | 162 (2.1) |  | 5247 (67.1) | 1420 (18.2) | 659 (8.4) | 263 (3.4) | 229 (2.9) |
| 1. Diarrhea | 5643 (72.2) | 1080 (13.8) | 676 (8.6) | 252 (3.2) | 167 (2.1) |  | 5814 (74.4) | 1116 (14.3) | 533 (6.8) | 200 (2.6) | 155 (2.0) |
| 1. Weakness | 4066 (52.0) | 1593 (20.4) | 1211 (15.5) | 574 (7.3) | 374 (4.8) |  | 4234 (54.2) | 1629 (20.8) | 980 (12.5) | 505 (6.5) | 470 (6.0) |
| 1. Insomnia | 5733 (73.3) | 1113 (14.2) | 595 (7.6) | 236 (3.0) | 141 (1.8) |  | 5876 (75.2) | 1094 (14.0) | 522 (6.7) | 199 (2.5) | 127 (1.6) |
| 1. Gloomy | 6140 (78.5) | 822 (10.5) | 504 (6.4) | 195 (2.5) | 157 (2.0) |  | 6241 (79.8) | 806 (10.3) | 406 (5.2) | 170 (2.20 | 195 (2.5) |
| 1. Irritability | 4147 (53.0) | 1533 (19.6) | 1108 (14.2) | 547 (7.0) | 483 (6.2) |  | 4385 (56.1) | 1610 (20.6) | 851 (10.9) | 455 (5.8) | 517 (6.6) |
| 1. Nervousness | 4829 (61.8) | 1263 (16.2) | 892 (11.4) | 422 (5.4) | 412 (5.3) |  | 4978 (63.7) | 1326 (17.0) | 752 (9.6) | 332 (4.2) | 430 (5.5) |

**Supplementary Table 4** Distribution of frequency of the Questions in PHQ-9.

|  | Frequency (percentage) | | | |
| --- | --- | --- | --- | --- |
|  | Not at all | Several days | More than half the days | Nearly every day |
| 1. Little interest or pleasure in doing things | 3347 (42.8) | 3573 (45.7) | 584 (7.5) | 314 (4.0) |
| 1. Feeling down, depressed, or hopeless | 4408 (56.4) | 2708 (34.6) | 481 (6.2) | 221 (2.8) |
| 1. Trouble falling or staying asleep, or sleeping too much | 4527 (57.9) | 2468 (31.6) | 477 (6.1) | 346 (4.4) |
| 1. Feeling tired or having little energy | 3337 (42.7) | 3502 (44.8) | 622 (8.0) | 357 (4.6) |
| 1. Poor appetite or overeating | 4139 (52.9) | 2999 (38.4) | 446 (5.7) | 234 (3.0) |
| 1. Feeling bad about yourself—or that you are a failure or have let yourself or your family down | 3960 (50.7) | 2802 (35.8) | 643 (8.2) | 413 (5.3) |
| 1. Trouble concentrating on things, such as reading the newspaper or watching television | 4428 (56.6) | 2693 (34.4) | 419 (5.4) | 278 (3.6) |
| 1. Moving or speaking so slowly that other people could have noticed? Or the opposite—being so fidgety or restless that you have been moving around a lot more than usual | 5331 (68.2) | 1988 (25.4) | 325 (4.2) | 174 (2.2) |
| 1. Thoughts that you would be better off dead or of hurting yourself in some way | 6204 (79.4) | 1233 (15.8) | 229 (2.9) | 152 (1.9) |
